# Shipping and storage temperature logger datasets for extended shelf life vacuum packaged chilled beef in the Chinese supply chain

**DOI:** 10.1016/j.dib.2019.104586

**Published:** 2019-09-28

**Authors:** Damian Frank, Yimin Zhang, Xin Luo, Xue Chen, Glen Mellor, Janet Stark, Joanne Hughes

**Affiliations:** a CSIRO, Agriculture and Food, Australia; b Shandong Agricultural University, Tai'an, Shandong Province, China

**Keywords:** Vacuum packaged chilled beef, Storage temperature

## Abstract

This article contains temperature logger datasets obtained for refrigerated cartons of Australian vacuum packaged chilled beef stored under near ideal conditions (∼- 1 °C) in Australia (CONTROL) and shipped via land and sea to three destinations in China (China-1, China-2, China-3) described in detail previously [1]. Cartons were stored for 84, 98, 120 and 140 days postslaughter. Temperature data were acquired during shipping and storage using i-buttons (Thermocron-TCS, Baulkham Hills, Australia) and LogTags (TRIX-16, LogTag Australia, Baulkham Hills, Australia).

**Table:** Specifications Table

Subject area	*Agricultural and biological sciences*
More specific subject area	*Refrigerated storage*
Type of data	*Microsoft Excel Worksheet*
How data was acquired	*Temperature data were acquired using i-buttons (Thermocron-TCS, Baulkham Hills, Australia) and LogTags (TRIX-16, LogTag Australia, Baulkham Hills, Australia) throughout shipping from the Port of Brisbane (Australia) to Shanghai International Port (Shanghai, China) and different destinations in China.*
Data format	*Raw*
Experimental factors	*Temperature monitoring of vacuum packaged chilled beef in cartons during shipping and storage under ideal conditions (CONTROL ∼ - 1.0 C°) and to three Chinese destinations for 84, 98, 120 and 140 days postslaughter.*
Experimental features	*Temperature data were acquired every* 25 min by *i-buttons and every* 15 min by *the LogTags throughout the storage period.*
Data source location	*CONTROL samples (CSIRO, Brisbane, Australia), Chinese samples various locations between Brisbane (Australia) and Tai'an (Shandong Province, China)*
Data accessibility	*Data is with this article*
Related research article	D. Frank, Y. Zhang, Y. Li, X. Luo, X. Chen, M. Kaur, G. Mellor, J. Stark, J. Hughes. Shelf life extension of vacuum packaged chilled beef in the Chinese supply chain. A feasibility study, Meat Sci., 153, 2019, 135–143 [1].

**Table:** 

**Value of the Data** • *Data can be used to understand typical temperature variations in vacuum packaged chilled beef in Chinese export cold supply chains* • *The data may be used in beef shelf life prediction models* • *Data allows a comparison of the performance of i-buttons and LogTag temperature loggers*

## 1 Data

Data is provided in spreadsheet format. Data for i-button and LogTag temperature loggers from cartons of chilled beef stored for various times with date and time annotation are provided and correspond to the study reported in Ref. [1]. Australia is a large exporter of vacuum packaged chilled beef (VPCB) and China is an increasingly important destination. Information regarding temperature variability that occurs during transport and storage of VPCB is important to estimate and model realistic shelf life limits. This article contains Microsoft Excel Worksheets (Supplementary 1) containing temperature logger data obtained from cartons of VPCB stored under ideal conditions (∼-1.0 °C) stored in Australia (CONTROL) and after land and sea transport to three destinations in China; China-1 (Near Shanghai), China-2 (Near Shanghai) and China-3 (Tai'an, Shandong Province, China) for samples stored for 84, 98, 120 and 140 days.

## 2 Experimental design, materials, and methods

Export quality Australian beef striploins were vacuum packaged according to a published experimental design [1]. VPCB was placed into cartons and temperature data were measured using i-buttons (Thermocron-TCS, Baulkham Hills, Australia) and LogTags (TRIX-16, LogTag Australia, Baulkham Hills, Australia). Data were acquired approximately every 25 min by i-buttons and every 15 min by the LogTags throughout the storage period. The time point of each temperature reading was recorded by the data loggers. VPCB was stored under near ideal conditions (∼- 1 °C) in Australia (CONTROL) and shipped via land and sea to three destinations in China (China-1, China-2, China-3). Cartons were stored for 84, 98, 120 and 140 days. The data were downloaded for each logger and imported into Microsoft Excel Worksheet format.
